# Shallow-Water Bryozoan Communities in a Glacier Fjord of West Svalbard, Norway: Species Composition and Effects of Environmental Factors

**DOI:** 10.3390/biology12020185

**Published:** 2023-01-26

**Authors:** Olga Yu. Evseeva, Alexander G. Dvoretsky

**Affiliations:** Murmansk Marine Biological Institute (MMBI), Russian Academy of Sciences (RAS), 183010 Murmansk, Russia

**Keywords:** Bryozoa, biodiversity, biomass, intertidal zone, driving factors, Barents Sea, Svalbard, Grønfjorden

## Abstract

**Simple Summary:**

Bryozoans are predominantly short-lived sessile organisms inhabiting both living and dead hard substrates. This group is considered a good biological indicator of environmental conditions. The Svalbard Archipelago is located at high latitudes, and the local benthic communities are affected by severe environmental conditions. In general, the bryozoan fauna in this region has been well studied, albeit to varying spatial degrees. Information regarding Grønfjorden is scarce and, therefore, we aimed to study species composition, diversity, and biomass of bryozoans in this glacier fjord. We found a rather diverse fauna with low biomasses for all species. Cluster analysis distinguished two groups of stations: one with lower diversity and biomass in warmer water, and the other with higher values in colder water. The most important factors shaping the bryozoan communities were water temperature and the proportional occurrence of macrophytes. Taking into account ongoing climate change, our results may be useful for further monitoring the effects of elevated temperatures on benthic organisms in the Arctic.

**Abstract:**

Despite significant research efforts focused on benthic assemblages in West Spitsbergen, there is a lack of knowledge regarding the shallow water bryozoan communities in Grønfjorden, a glacier fjord belonging to the Isfjorden system, Norway. Here, we studied species composition, richness, distribution, and biomass of bryozoans in the intertidal and upper subtidal zones of Grønfjorden in summer. We found 62 bryozoan species, among which *Celleporella hyalina* (Linnaeus, 1767), *Harmeria scutulata* (Busk, 1855), and *Tegella arctica* (d’Orbigny, 1853) were most prevalent while the highest contributions to the total biomass were registered for *Eucratea loricata* (d’Orbigny, 1853), *Tricellaria gracilis* (Van Beneden, 1848), *Turbicellepora incrassata* (Lamarck, 1816), and *Tricellaria ternata* (Ellis and Solander, 1786). Alpha-diversity varied from 1 to 50 averaging 15.1 ± 2.6 species. Bryozoan biomass ranged from 0.008 to 10.758 g m^−2^ with a mean value of 2.67 g m^−2^ being lower than in the central and northern parts of the Barents Sea. For the first time, we registered the presence of the circumpolar bryozoan *Amathia arctica* in Svalbard waters probably as a result of stronger advection of Atlantic water into the fjord. Cluster analysis revealed two groups, mainly composed of stations in colder and warmer waters. A relatively high proportion of outlying stations reflected habitat heterogeneity in Grønfjorden. Redundancy analysis indicated that bryozoan diversity and biomass were strongly negatively associated with temperature. A positive relationship was found between bryozoan biomass and the proportional contribution of macrophytes to a pool of substrates. Our study provides a reference point for further monitoring of changing marine ecosystems at high latitudes.

## 1. Introduction

Biological and chemical processes in the Arctic Ocean and its marginal seas are currently being affected by fluctuations in sea ice cover, warmer air and ocean temperatures, and associated changes in ocean currents [1]. In the Barents Sea Large Marine Ecosystem, the effects of climate forcing are especially pronounced on the major inflow shelves, where northward currents from the Atlantic transfer nutrients and plankton into the Arctic [2,3,4]. The Barents Sea and the Fram Strait region are the major gateway into the Arctic forming a transition zone between sub-Arctic and Arctic marine ecosystems [5,6]. This highly productive area provides important ecosystem services; supports large cod, haddock, saithe, capelin, northern shrimp, red king crab, and snow crab stocks [7,8,9,10,11,12]; and contributes to the development of polar aquaculture [13,14,15].

Arctic waters are characterized by a relatively low diversity of zooplankton [16,17,18,19] and fish [20] while benthic fauna is richer and comprises more than 90% of marine animals in the Arctic [21]. Bryozoans are colonial, sessile, filter-feeding benthic invertebrates [22], which often dominate epibenthic assemblages on the lower surfaces of hard substrates both of natural (living or dead) and artificial origin [23,24,25,26,27,28,29,30]. Most bryozoans brood their embryos and release short-lived lecithotrophic larvae [31]. Being typically attached organisms, bryozoans exhibit two groups of behavioral reactions: the work of cilia (excepting some male autozooidal polymorphs without cilia) and different tentacle movements and entire polypide of the zooid performed independently of the activities of other colony members. These activities can be associated either with feeding and cleaning or with reproduction processes [32]. Bryozoans are considered one of the most species-rich and diverse groups in terms of body size, structure, form, and autecological traits [23,31,33,34]. In the Barents Sea, the diversity of bryozoans is highest among all Arctic regions and comprises 328 species [25]. Because of their sessile lifestyle, intimate association with the substrates, and varied life-history traits, bryozoans are highly sensitive to fluctuations in environmental conditions and accurately reflect potential impacts on habitat quality of a given location [35,36]. Thus, this group may be considered a good biological indicator of environmental conditions [37].

The archipelago of Svalbard is situated between 76° and 80° N and is bordered by the Arctic Ocean to the north, the Barents Sea to the south and east, and the Norwegian Sea to the west. The Svalbard region is a transition zone between warm and saline Atlantic water mass and cold and less saline Arctic water mass [10,38]. These water masses provide a driving force for controlling the distribution and functioning of unique ecosystems, the study of which can provide significant insights into the role of climate in driving the structure and species composition of benthos in general [39] and bryozoan communities in particular [24]. There have been a lot of studies focused on bryozoan diversity in relation to environmental factors in Svalbard waters, but the majority of comprehensive investigations were focused on Kongsfjord [24,40,41,42,43,44,45,46]. Only a few studies have been undertaken in other areas of the archipelago [43,47,48,49,50], but the bryozoan fauna of Grønfjorden (West Svalbard), especially in shallow waters, has not yet been well studied.

The aim of our research is to describe the diversity and spatial distribution of bryozoans in the intertidal zone and at shallow-water sites of Grønfjorden and evaluate the most important environmental factors that affect the structure and biomass of bryozoan communities in the study area.

## 2. Materials and Methods

### 2.1. Study Area

**Figure 1:**
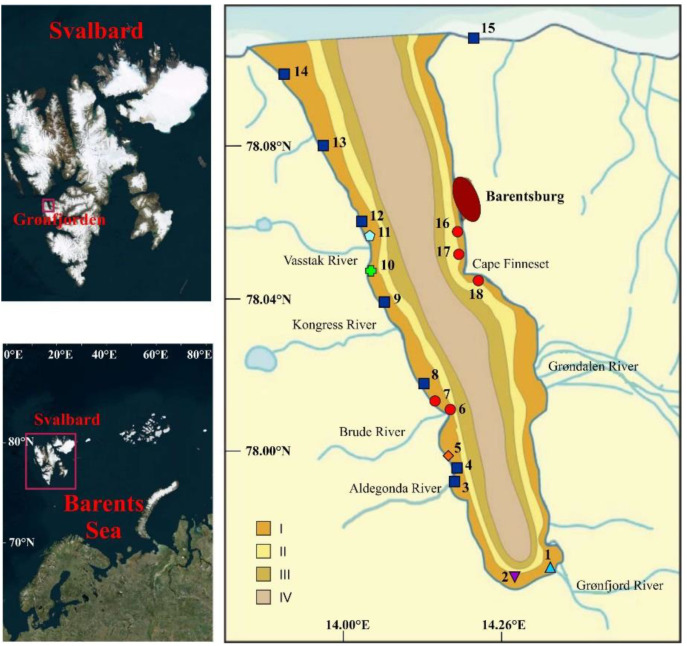
Location of sampling stations and sediment composition in Grønfjorden, West Spitsbergen. Sediments: I—Gravel, pebble, and boulders; II—sand; III—silt; IV—clay [52]. Groups of stations based on bryozoan biomass: red—Cluster 1, blue—Cluster 2, other colors—outliers.

Grønfjorden (also cited as Grøn-fjord, Gren-fjord, or Grenfjord) is located on the southwestern coast of West Spitsbergen between 77°07′ and 77°58′ N and 13°56′ and 14°20′ E. The territory with an area of more than 193 km^2^ is the southwestern branch of Isfjorden, having a shape elongated in the meridian direction (length 16.3 km, width 2.0–6.4 km, maximum depth 132 m). The length of the coastline is 36.9 km [51]. The eastern coast receives freshwater input from the Grøndalen River, southern areas are characterized by the impact of the Grønfjord River and Bretjern Creek, and, over the western margin, freshwater discharges from the Aldegonda, Brude, and Kongress rivers as well as from some creeks inject large volumes of nutrient-rich waters and organic matter into the fjord [52]. The oceanography of the area is influenced by saline and relatively warm Atlantic water transported northward along the shelf break by the West Spitzbergen Current. As a result, local temperatures are higher than usually recorded at these latitudes. The lowest air temperatures are registered in February (average value −18 °C) while the warmest month is July (8 °C) [53]. The lowest temperature value in the surface water layer (−1.8 °C) occurs in February and the highest (7.5 °C) in late July–August. Summer bottom temperatures range from 1.0 to 7.5 °C [52]. Minimum salinity (4 psu) is associated with a high input of freshwater at river confluences, while maximum salinity (35 psu) is registered in the lower water layers [54]. Although extensive multiyear variations in ice cover have been recorded in Grønfjorden, ice coverage usually starts to appear in mid-December, and maximum ice extent occurs in March–April and lasts until mid-May. Ice thickness does not exceed 60 cm [52]. Sediment composition demonstrates clear bathymetric zonation; boulders, pebbles, and gravel occur in the upper littoral zone, these sediments are replaced with sand in the lower littoral, and at deeper sites, thin-grained sediments (silt and clay) are predominant (Figure 1).

The long-term average annual wind velocity is 3.1 m s^−1^, with the main directions from the northwest, north, and south during the summer period, and turning southeast during the winter season. Calms are observed here more often from January to February [52]. Precipitation predominantly falls as snow (563 mm per year), with maximum levels being registered in December (62.2 mm) and January (59.5 mm). The mean relative humidity is 78% [52].

### 2.2. Sampling and Processing

A total of 18 stations was sampled for bryozoans in July 2014 and 2015 (Figure 1). At each sampling location, water samples were collected, and seawater temperature and salinity were determined using portable profilers. Substrates were classified as follows: clay (particle size < 0.002 mm), silt (0.002–0.02 mm), sand (0.02–1 mm), gravel (1–10 mm), pebble (10–100 mm), boulders (>100 mm), and macrophytes. Sediment characteristics were obtained from Meshcheryakov [52]. The contribution of macroalgae to the pool of substrates was determined from digital high-quality photographs using ImageJ software, version 1.53 [55].

Sampling methods differed according to depth and sediment composition. At each intertidal station (*n* = 8; Stations 1, 3, 4, 5, 7, 11, 13, and 15), bryozoans were collected by hand in triplicate from a 50 × 50 cm quadrate placed on hard substrates. Nearshore stations (up to 10 m) located on soft bottoms with pebbles and gravel (*n* = 5; Stations 8, 9, 12, 14, and 18) were sampled using a hand dredge, consisting of a 20 × 53 cm steel frame with a digging blade, towed along 60 m transects perpendicular to the shore. On the coast, these samples were sorted for substrates covered with bryozoans. Station 2 located at the head of the fjord (depth 15 m) was sampled using a 0.03 m^2^ Petersen grab. Finally, some stations at 5–20 m (*n* = 4; stations 6, 10, 16, and 17) were sampled by divers using a 25 × 25 cm metal frame.

The collected material was fixed in 4% buffered formalin. In the laboratory, bryozoans were identified under an MBS-10 stereomicroscope using the comprehensive monographs of Kluge [33,34] and more recent literature. The diversity of bryozoan communities was estimated using the Shannon index [56] calculated from the species biomass and Pielou’s evenness index [57]. The Chao2 estimator was calculated to estimate the maximum expected number of bryozoan species in the study area. Chao2 estimates the asymptote of the species accumulation curve by taking into account the effect of rare species on the total richness and may provide a better estimate of true species richness for small numbers of samples [58].

### 2.3. Statistical Analysis

Bryozoan community analysis was performed using multivariate statistics in the software package PRIMER 5.0. Diversity indices were square-root-transformed, and biomass was fourth-root-transformed to decrease the weight of dominant species. Cluster analysis was used to distinguish the spatial communities based on the Bray–Curtis similarity measure and group average linkage method. A non-metric multidimensional scaling (nMDS) ordination based on the Bray–Curtis similarity matrix was used to visualize the compositional similarity of samples. The significance of differences in the bryozoan communities between station groups based on hierarchical clustering was assessed using a one-way analysis of similarities (ANOSIM), in which global R = 0 indicates no separation of groups, and global R = 1 indicates a complete separation [59]. To determine whether sampling procedures influenced station clustering, we also performed ANOSIM among stations sampled by different methods. The similarity percentage (SIMPER) procedure, based on the Bray–Curtis similarity matrix, was used to identify those species contributing most to the dissimilarities in the station groups [59].

Differences in bryozoan biomasses and diversity indices as well as in environmental variables between the station groups were quantified with a one-way analysis of variance (ANOVA) if the data were normally distributed and in the case of variance homogeneity. In the case of non-normal distributions or variance heterogeneity, the Kruskal–Wallis test was conducted. Principal component analysis (PCA) was also used to discern patterns in environmental variables at our sampling stations. Before PCA, all variables were standardized (x′) using the following formula: x′ = (x − m)/SD, where x is the non-transformed value, m is the overall mean for the data set, and SD is the standard deviation.

Redundancy analysis (RDA) was performed to evaluate relationships between local environmental variables and diversity and biomass of bryozoans. RDA was selected because detrended correspondence analysis showed that the length of the first gradient for the bryozoan communities was lower than three, indicating a linear variation [60]. The input dataset of environmental variables included water temperature, salinity, depth, and type of substrate. Two datasets were used for response variables: the first included biomasses of all species plus the total biomass; and the second included the number of species (total and calculated for different construction forms and orientation of colonies), H′, and J′. All data were square-root-transformed in the case of diversity RDA and fourth-root-transformed in the case of biomass RDA. A Monte Carlo permutation test (*n* = 999) was used to reveal the environmental variables that best explained the bryozoan biomass and diversity data. Before including the selected environmental variables in the RDA, the data were tested for collinearity, and the variables that had a variance inflation factor higher than 10 (VIF > 10) were excluded. All ordinations were performed in CANOCO for Windows v. 4.5 [60]. Mean values were presented with standard errors.

## 3. Results

### 3.1. Environmental Conditions

**Figure 2:**
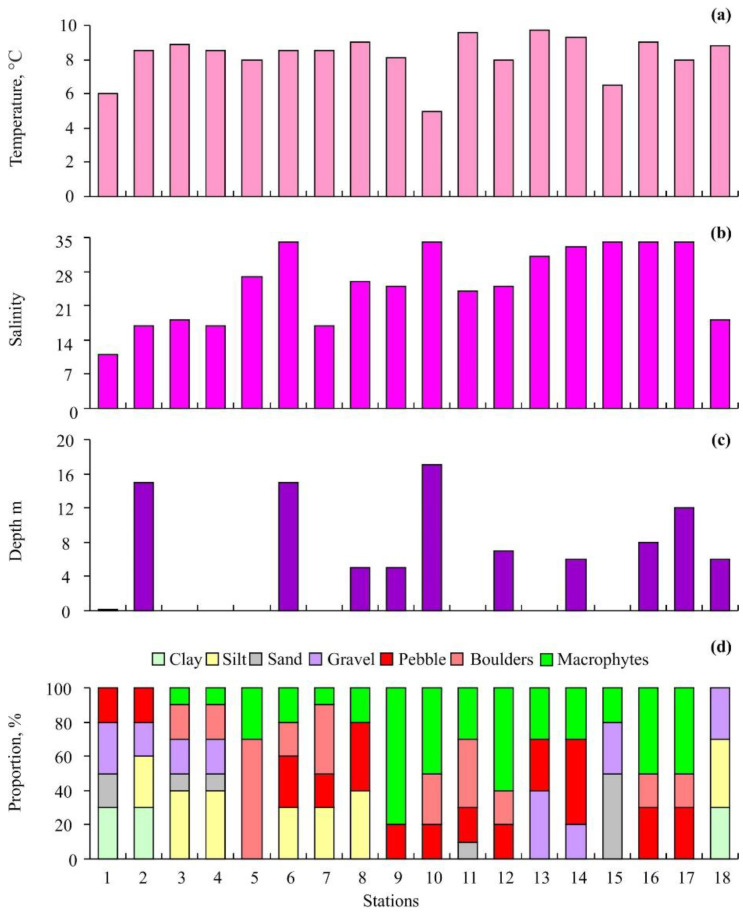
Environmental conditions at sampling stations in Grønfjorden: (**a**)—temperature, (**b**)—salinity, (**c**)—depth, (**d**)—substrates.

The lowest water temperatures were detected at Station 10 (5.0 °C) located in the middle part and Station 1 (6.0 °C) at the head of the fjord, while the highest values were registered at Stations 18 (10.1 °C) and 13 (9.7 °C) (Figure 2a).

Decreased salinity levels (11–18 psu) were found at Stations 1 and 2 located at the head of the fjord, Stations 3 and 4 at the mouth of the Aldegonda River, Station 7 located to the north of the Brude River, and Station 18 in the vicinity of Cape Finneset (Figure 2b). Stations 2, 6, 10, and 17 were sampled at depths ranging from 12 to 17 m, while other stations covered the littoral zone and depths <6 m (Figure 2c). Clay was found at Stations 1, 2, and 18. The highest silt content was registered at Stations 3, 4, and 8. Station 15 was characterized by the highest sand content (Figure 2d). Gravel was present at Stations 1–4 located in the inner part of the fjord, at Stations 13–15 located at the fjord entrance, and at Station 18. Pebbles, boulders, and macroalgae occurred at most stations in different combinations (Figure 2d).

**Figure 3:**
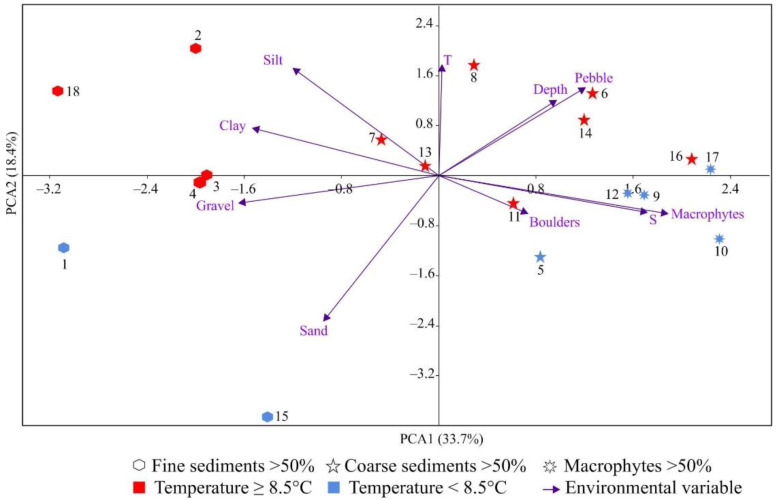
Biplot of principal component analysis of environmental variables at sampling stations located in Grønfjorden. T—temperature (°C), S—salinity, Depth—depth (m), Clay, Silt, Sand, Gravel, Pebbles, Boulders, Macrophytes—proportions of corresponding substrates (%).

The first four principal components with eigenvalues >1 accounted for 81.8% of the total variance. Sampling sites were separated along PC1 according to sediment composition, with stations located on fine-grained sediments generally having negative PC1 scores and stations located on coarse-grained sediments and macrophytes generally having positive PC1 scores (Figure 3).

The second axis separated our sampling stations mostly according to thermal regime. Stations located in warmer water had positive PC2 scores, whereas stations located in colder water had negative PC2 scores (Figure 3).

### 3.2. Bryozoan Diversity, Biomass, and Community

A total of 62 species of bryozoans belonging to 2 classes, 3 orders, 29 families, and 37 genera was identified in Grønfjorden (Table 1).

The most diverse order was Cheilostomatida (46 species, 74.2%), followed by Cyclostomatida (11 species, 17.7%) and Ctenostomatida (5 species, 8.1%). Calloporidae was the most diverse family (9 species, 14.5%) followed by Bryocryptellidae, Bugulidae, and Candidae (each accounted for 4 species, 6.5%). Bryozoans were found attached to solid substrates including the brown seaweed *Saccharina latissima*, and at Sites 13 and 15, bryozoans also colonized dead thalli of this macrophyte.

Biogeographic affinity of bryozoans found in Grønfjorden indicated the predominance of Boreo-Arctic species (43 species, 69.4%). The proportions of Arctic and boreal species were 22.9% (14 species) and 8.1% (5 species), respectively. With regard to construction forms, flexible bryozoans (5 species, 8.1%) were much less frequent than calcified ones (57 species, 91.9%), and with respect to orientation of colonies, the proportion of encrusting forms (44 species, 71%) was 2.5 times higher than that of erect bryozoans (18 species, 29%).

**Figure 4:**
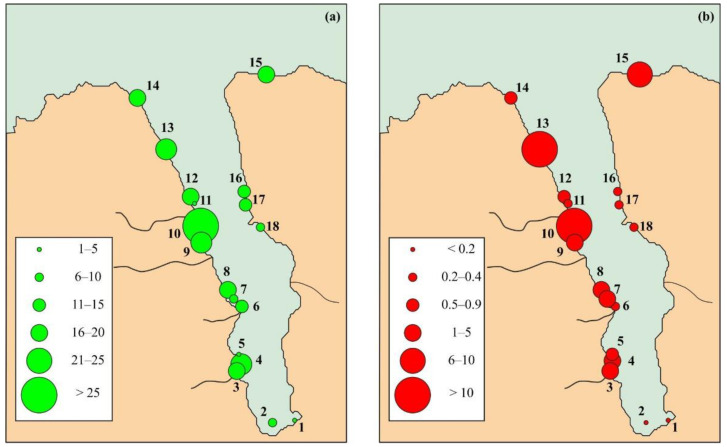
Variations in bryozoan species richness (**a**) and biomass (**b**), g m^−2^, in Grønfjorden.

Twenty-one species (33.9%) were found at single stations. The most frequent species were *Celleporella hyalina* (16 stations), *Harmeria scutulata* (15), *Tegella arctica* (15), *Callopora craticula* (14), and *Microporella arctica* (14). Minimal species richness (SR) was found at Stations 5 (1 species) and 11 (2 species), while the maximum was recorded at Station 10 (50 species) (Figure 4a).

The highest values of H′ (3.36) and J′ (0.822) were found at Station 14. Mean values for SR, H′, and J′ were 15.1 ± 2.6, 1.81 ± 0.22, and 0.459 ± 0.053, respectively. The Chao2 index was calculated to be 80, indicating that we sampled 78% of the estimated taxa pool.

Bryozoan biomass varied in a wide range throughout the study area (Figure 4b), with minimum values (0.008 and 0.043 g m^−2^) being registered at the head of the fjord (Stations 1 and 2, respectively), and maximum values occurred at Stations 10 (10.938 g m^−2^), 13 (10.758 g m^−2^), and 15 (6.044 g m^−2^). Overall, 95% of the total biomass was provided by 18 species, among which the highest contributions to the total material were found for *Eucratea loricata* (27.5%), *Tricellaria gracilis* (12.5%), *Turbicellepora incrassata* (9.1%), *Tricellaria ternata* (9.1%), *Alcyonidium hirsutum* (7.0%), *Crisiella producta* (5.8%), and *Celleporella hyalina* (4.4%). The average bryozoan biomass in Grønfjorden was calculated to be 2.67 ± 0.82 g m^−2^.

**Figure 5:**
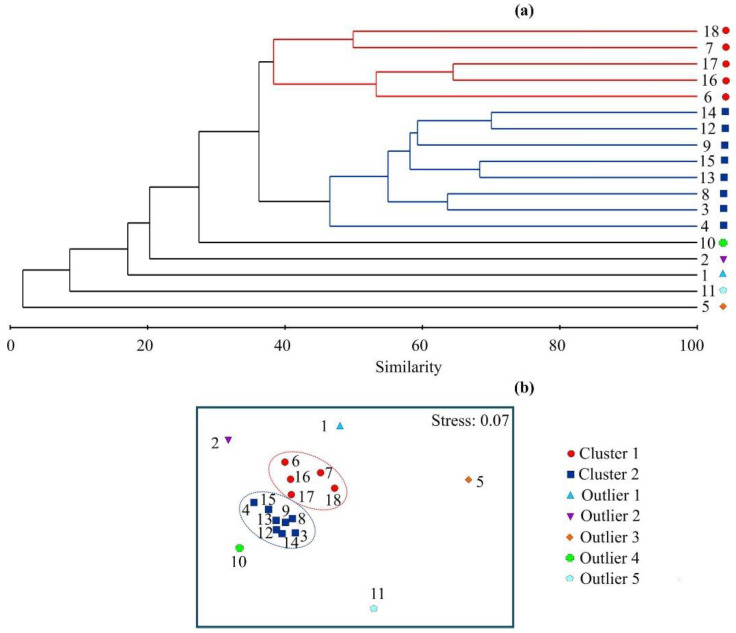
Dendrogram resulting from clustering (**a**), and a 2D nMDS plot (**b**) summarizing the similarity of bryozoan fauna in Grønfjorden performed on the Bray–Curtis similarity matrix produced from fourth-root-transformed biomass data.

Cluster analysis of the bryozoan community composition and biomass revealed two groups at a 36% similarity level (Figure 5a), separating Stations 6, 7, 16, 17, and 18 (Cluster 1) from Stations 3, 4, 8, 9, 12, 13, 14, and 15 (Cluster 2).

The remaining five stations with extremely low (Stations 1, 2, 5, and 11) and high (Station 10) diversity and biomass were recognized as outliers. The nMDS showed distinct, isolated separation of these station groups (Figure 5b), supporting the results of clustering. Stations from Cluster 1 had significantly higher clay content than stations from Cluster 2 (ANOVA, F = 10.39, *p* = 0.004) but a significantly lower proportion of macrophytes (ANOVA, F = 7.44, *p* = 0.012). Stations belonging to Cluster 1 were located in warmer water (8.0 to 10.1 °C) than stations grouped in Cluster 2 (6.2 to 9.7 °C), but the low sample size did not allow us to identify a significant difference between the mean values (*p* > 0.05). Nevertheless, the separation of our sampling stations in relation to water temperature was confirmed by PCA (Figure 3).

One-way ANOSIM revealed significant differences in the bryozoan community composition among the groups delineated with the cluster analysis (global R = 0.883, *p* = 0.010). Accordingly, Cluster 1 was significantly different from Cluster 2 (R = 0.726, *p* = 0.010). No significant variation was apparent when sampling methods (hand collection, dredging, and SCUBA) were used as categorical variables in ANOSIM (global R = 0.077, *p* = 0.752). The SIMPER analysis revealed that the average similarity within stations in Cluster 1 was 45.1%, and within stations in Cluster 2 it was 55.2%. The average dissimilarity between the two clusters was 63.8%, with 10 species contributing >50% to the total dissimilarity (Table 2).

Significantly higher biomasses of *Cribrilina spitzbergensis*, *Eucratea loricata*, *Microporella arctica*, and *Tricellaria ternata* were found at stations in Cluster 2 (*p* < 0.05). The total bryozoan biomass at these stations was almost five times higher than at stations in Cluster 1 (3.984 ± 1.172 vs. 0.838 ± 0.598 g m^−2^, Kruskal–Wallis test, H = 5.49, *p* = 0.019). SR also differed significantly between Cluster 1 (range 7–15, mean 11 ± 1 species) and Cluster 2 (16–23, 19 ± 1 species) (ANOVA, F = 28.00, *p* < 0.001).

### 3.3. Relationships between Bryozoan Data and Environmental Variables

**Figure 6:**
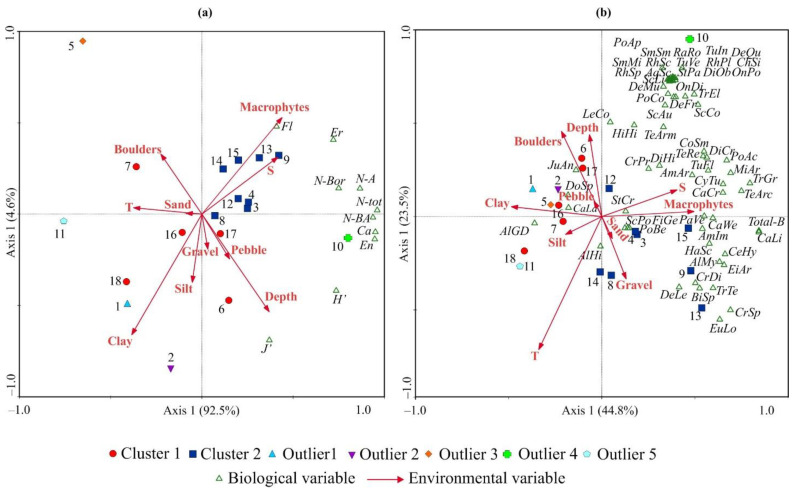
Ordination triplots of samples by redundancy analysis with respect to bryozoan diversity (**a**) and biomass (**b**) and their relations to environmental variables in Grønfjorden. The proportions of the total variability explained by the first two axes are given. Biological variables: Biomass for each species (for species abbreviations see Table 1), Total-B—total biomass, Ca—number of calcified bryozoans, Fl—number of flexible bryozoans, En—number of encrusting bryozoans, Er—number of erect bryozoans, N-Bor—number of boreal species, N-BA—number of Boreo-Arctic species, N-A—number of Arctic species, N-Tot—total number of species, En—number of encrusting species, Er—number of erect species, Ca—number of calcified species, Fl—number of flexible species, H′—Shannon index, J′—Pielou’s evenness. Environmental variables: T—temperature (°C), S—salinity, Depth—depth (m), Clay, Silt, Sand, Gravel, Pebbles, Boulders, Macrophytes—proportions of corresponding substrates (%).

The RDA based on diversity indices of the bryozoan fauna showed that the first axis explained 92.5% of the total variation. This axis of the RDA plot (Figure 6a) indicated a clear separation between stations with high (right side) and low (left side) species diversity.

Moreover, the upper right quadrant was characterized by all stations belonging to Cluster 2. The second axis of the RDA plot explained 4.5% of the total variation and separated stations according to living forms of bryozoans with a higher occurrence of flexible and erect species on the upper side and higher SR of calcified and encrusting forms on the lower side of the RDA plot. A forward selection procedure (Monte Carlo permutations test) revealed that only water temperature was the factor that contributed significantly to the RDA model (Table 3).

The RDA based on bryozoan biomasses indicated that the first two axes explained a large proportion of the variance in the data (RDA 1 = 44.8% and RDA 2 = 23.5%). The ordination triplot showed that the first axis was closely negatively correlated with water temperature and positively with the proportion of boulders and depth, while the second axis was positively correlated with the proportion of macrophytes and strongly negatively correlated with temperature (Figure 6b). Most bryozoan species were positively correlated with both axes, indicating that their biomasses tended to increase at sites with higher portions of boulders and macrophytes and lower water temperatures. The RDA 1 axis clearly separated sampling sites with low (left side) and high (right side) biomass, with all stations of Cluster 2 except for Station 12 being located in the lower right quadrant. The forward selection procedure found that the most significant environmental factors explaining the variance in the bryozoan taxa biomasses were water temperature and the proportion of macrophytes (Table 3).

## 4. Discussion

### 4.1. Environmental Conditions

Grønfjorden is characterized by a typical estuarine circulation regime. There is an inflow of saline water from the sea with the main current direction being towards the head of the bay along the western coast. The current then reaches the inner part of the bay and outflows into the Isfjorden area along the eastern coast [61]. This denser saline water enters below the low-salinity layer, which is formed by the fresh water from the local rivers running seaward in the upper layer and then entraining salt water from below and carrying it out of the fjord [62]. This circulation pattern is complicated over short time periods by the periodic effects of tidal flow and aperiodic modifications caused by strong winds [63]. The lowest water temperatures were found for Stations 1 and 10, probably because these locations were affected by cooled freshwater from local glaciers. Station 15 was located in the outer zone impacted by cold oceanic water, which explains the low temperature and high salinity at this site. Other stations with true marine conditions were located at a sufficient distance from the regions affected by freshwater run-offs where water salinity was low (Stations 1–4, 7, and 8). Since water salinity increases with an increase in depth [63], almost all stations located below 5 m had normal salinity (Figure 2).

In Grønfjorden, coastal accumulation of sediments occurs mainly in river and glacier valleys, where decreased wave movement activity and regular loads of terrigenous material exported from river discharges promote intense sedimentation processes [52]. As a result, most stations located near the mouths of large rivers emptying into the fjord (Stations 1–4, 6–8, and 18) had an increased content of fine sediment particles. In addition, mean outflow of the upper layer in the fjord results in the drift of flotsam and silt [61]. For this reason, fine sediments were widely present throughout the study area. PCA also confirmed separation of our sampling stations in relation to the thermal regime, sediment composition, and occurrence of macrophytes (Figure 3). Thus, Grønfjorden is an estuarine area with a wide range of habitats combining both favorable and less favorable conditions for bryozoans.

### 4.2. Bryozoan Diversity, Biomass, and Communities

Estuarine regions that are affected by significant freshwater discharges, with gradients in environmental conditions, have high biological activity and diversity due to a wide range of ecological niches [64,65,66]. For this reason, despite our study being restricted to a relatively low number of sampling stations, we registered high species richness of the bryozoan fauna in Grønfjorden. Moreover, according to the estimated Chao2 index, our sampling campaign was able to sufficiently survey the entire bryozoan biodiversity of the region, where 18 additional taxa may theoretically be discovered or reported. However, Gulliksen et al. [47] revealed the presence of 181 species in Svalbard waters. Gontar et al. [41] working in Kongsfjorden found 101 species and updated the list of the previous authors with 96 new records using both their own and published data. Kuklinski [42] recorded 123 bryozoan species in Kongsfjorden and later described three new species in collaboration with colleagues [67,68]. A list of species by Palerud et al. [43] covering all waters around the archipelago included 182 species. Our list of bryozoans contains 62 species, thus accounting for 34% of the total diversity, and it is unlikely that most of the 18 additional taxa predicted by the Chao2 index will be new for the bryozoan fauna of Grønfjorden. The lower number of bryozoan species registered in shallow waters of Grønfjorden is expected, because in the Barents Sea, the most diverse bryozoan fauna is registered at depths between 50 and 100 m [23,69].

Our survey indicated the presence of one species that had not previously been reported in this region, the ctenostomatid flexible bryozoan *Amathia arctica* (Busk, 1880)*,* which is known to be an Arctic circumpolar species. It has previously been recorded in the White Sea, the Barents Sea including Franz-Josef land, the Kara Sea, and the Bering Sea [33,34,69,70,71]. Svalbard waters have suitable habitat conditions for the mentioned species, and this new record is not surprising, especially during the period of ongoing global warming when the stronger inflow of Atlantic water into the Barents Sea leads to shifts in local circulation patterns [72,73], including the West Spitsbergen shelf area [74], thus promoting larval drift and range expansion of aquatic animals [75].

Previous studies have shown substantial spatial variations in bryozoan biomasses throughout the Barents Sea. For example, in the central part, the mean biomass reached 13 g m^−2^ [76], while in the northeastern part, this value was as low as 1 g m^−2^ at most stations [77], and in the northern part, namely, in Tykhaya Bay of the Guker Island (Franz-Josef Land), a maximum of biomass amounted to 485 g m^−2^ [69]. These studies were conducted using a van Veen grab and covered deep-water sites. A recent study undertaken in the intertidal zone in the southern Barents Sea has shown a mean bryozoan biomass value of 2.25 ± 0.95 g m^−2^ [78], i.e., very close to the mean biomass we registered for the intertidal and shallow-water zones of Grønfjorden (2.67 ± 0.82 g m^−2^). We can hypothesize that the lower biomasses of bryozoans in the littoral zones of Grønfjorden and on the eastern coast of the Kola Peninsula in comparison to deeper waters of the Barents Sea may be attributed to more severe environmental conditions in these intertidal zones in terms of higher variability in temperature and salinity and much higher temperatures and much lower salinity at some sampling stations within these coastal sites in summer [69,76,78].

We found that different sampling methods did not bias our results, and, hence, data obtained using different sampling procedures are comparable. Kukliński [42], Kuklinski and Barnes [48], and Kuklinski et al. [24] also used different sampling techniques to study bryozoans in Svalbard waters and reported no effects of sampling on bryozoans. We found no distinct boundaries between the two types of bryozoan communities in our study area (Figure 1). Usually, a clear spatial distribution of benthic assemblages takes place along environmental gradients both in deep-water and shallow-water regions including gulfs and fjords [39,66,79,80,81]. In contrast to many other fjord areas [82,83], intertidal habitats in Grønfjorden are affected by multiple run-offs from rivers and creeks [52] that create habitat heterogeneity at neighboring locations and even within the same site (Figure 2). This habitat heterogeneity, most likely, is the main reason for the patchiness in the distribution of bryozoan communities in Grønfjorden.

### 4.3. Environmental Control of Bryozoan Communities

For bryozoan communities, depth and sediment characteristics have been established to be the most important driving factors in Svalbard [24,41,48]. However, the mentioned studies covered wider depth ranges, and the sampling efforts were not coupled with temperature and salinity measurements.

Our research was focused on the shallow-water zone, and, as expected, the RDA showed no associations between sampling depth and bryozoan diversity and biomass. In contrast, water temperature was the major determinant of bryozoan communities explaining 21 and 13% of the total variance in diversity and biomass, respectively (Table 3). The strong negative association between this factor and benthic community characteristics is explained by the predominantly cold-water nature of most bryozoan species found in Grønfjorden (Boreo-Arctic and Arctic taxa together accounted for 92.3% of the total number of bryozoan species). In contrast to mobile benthic species, which are able to escape unfavorable conditions, bryozoans, like other sessile taxa, have very limited opportunities to leave unfavorable habitats, either with drifting/terrigenous material [84] or with their mobile crab hosts [26,85,86,87]. Thus, when heatwave events occur, bryozoans have to expend more energy for thermoregulation and, in turn, to reduce the energy investment for growth and/or reproduction [88]. Over the past decades, various manifestations of warming have been registered in the Arctic, including rapid sea ice edge retreat, seasonal shifts in phytoplankton blooms, relatively warmer summer waters, and enhanced winter convection [89,90]. The Isfjorden system is also affected by the inflow of Atlantic water, and the local benthic communities, including those in Grønfjorden, are currently being affected by elevated temperatures and wind forcing [74]. The role of the thermal regime in shaping bryozoan communities of Grønfjorden is well illustrated by the results of our cluster analysis; SR and biomass of bryozoans were significantly higher at colder (Cluster 2) than at warmer stations (Cluster 1). A decrease in biomasses and diversity indices with increasing water temperature has also been reported for intertidal bryozoan communities on the eastern side of the Kola Peninsula [78].

In line with previous studies [24,41,48], we found that bryozoans tended to be more abundant on coarser sediments and macrophytes, but a significant contribution to the total RDA model was confirmed only for algae. At sites with a high content of thin-grained sediments (Stations 3, 4, 6, and 8), macrophytes became very important for bryozoans to settle on. Vegetated marine systems are known to provide coastal protection from erosion and flooding [91]. Thus, at most locations with significant water turbulence, laminarian kelps may be more desirable for bryozoans than gravel, pebbles, and boulders, because the macrophytes create microcosms relatively sheltered from storm events and disturbance caused by strong waves and tidal currents, which prevent successful settlement and may promote wave-related abrasion of bryozoan colonies. This is particularly true for flexible and erect bryozoans, which are usually less firmly attached to substrates than calcified and encrusting forms. Indeed, as can be seen in Figure 6a (right upper quadrant), the “Macrophytes” vector was very close to symbols indicating SR of the mentioned living forms. The same pattern, high abundances of erect bryozoans on macroalgae, has been reported by Eggleston [92] for sub-littoral benthic communities of the Isle of Man, Irish Sea.

The sampling stations included in Cluster 2 were characterized by higher diversity and biomass, and this result would also be attributed to a higher frequency of macroalgal occurrence here when compared to the stations from Cluster 2. The role of macrophytes in the survival of bryozoan colonies under harsh conditions of Svalbard fjords has been widely discussed in the literature [40,45,48].

Our RDA model explained 68.3% of the total variation in bryozoan biomass, indicating that other factors may be responsible for the biomass distribution across the study area. For example, riverine inputs not only lead to decreased salinity (a factor that reduced bryozoan biomass considerably [78] at Stations 1 and 2), but also to stronger sedimentation processes, and if the sedimentation rate is higher than the growth rate of a bryozoan, the colony cannot effectively ingest suspended particles and dies. This process, if any, seems to have higher significance for encrusting forms and may explain the lowest SR at Station 5, where the water temperature of 8 °C and high proportions of boulders and macrophytes would result in high diversity and biomass. Specific substrate properties can also alter bryozoan communities. For example, at Site 10, bryozoan biomass varied in a wide range, averaging 11.0 g m^−2^. The highest value (31.5 g m^−2^) was found at 20 m under favorable conditions in terms of predominance of coarse-grained substrates and low water temperatures (5 °C), while the lowest biomass (2.7 g m^−2^) was registered at a 10 m depth under the same temperature conditions but in a *Lithothamnion* community. Although the surface of this coralline alga is a hard substrate, our previous unpublished observations indicate that bryozoans demonstrate expressed avoidance responses to this type of substrate, probably due to its anti-fouling properties. This situation, when the presence of antagonist species (including other bryozoans) altered the bryozoan community structure, even if other environmental conditions were favorable, has previously been described in numerous publications [93,94,95,96].

## 5. Conclusions

The activity of tidal glaciers and significant freshwater inputs into the basin of Grønfjorden leads to high turbidity, high sedimentation rates, and salinity depressions. A variety of micro-habitat conditions resulting from the river run-offs and their interactions with the inflow of saline water from Isfjorden support a rather diverse bryozoan fauna in the shallow waters of the fjord. The average biomass was low, reflecting high variability in environmental conditions and elevated water temperatures, which is unfavorable for the local fauna composed of Boreo-Arctic and Arctic species. A more intense circulation due to higher current velocity and stronger winds associated with climate change may be responsible for a new distributional record of the ctenostomatid bryozoan *Amathia arctica* in the Svalbard Archipelago region. We revealed two bryozoan communities different in terms of diversity and biomass, with higher values at stations located in colder water and with a more frequent occurrence of macrophytes. Statistical analysis showed that water temperature was the most important driver of bryozoan diversity and biomass, with strong negative correlations between the input and output variables. Bryozoan biomasses were also positively associated with macrophytes, reflecting the importance of laminarian kelps in protecting bryozoans from wave disturbance and abrasion processes. Further monitoring is required to assess the effects of climate forcing on benthic communities in the Arctic.

## Figures and Tables

**Table 1:** Species composition and biomass of bryozoan species found in the intertidal and upper subtidal zones of Grønfjorden.

Taxa	Code	X	SE	Min	Max	Or	CF	OF	Stations
Cyclostomatida									
*Crisiella diversa* (Kluge, 1955)	CrDi	0.207	0.136	0.050	0.615	A	Ca	Er	12, 13, 14, 15
*Crisiella producta* (Smitt, 1865)	CrPr	0.936	0.902	0.003	2.740	BA	Ca	Er	4, 6, 10
*Diplosolen obelium* (Johnston, 1838)	DiOb	0.032	0.000	0.032	0.032	B	Ca	En	10
*Disporella crassiuscula* (Smitt, 1867)	DiCr	0.007	0.002	0.005	0.009	A	Ca	En	10, 13
*Disporella hispida* (Fleming, 1828)	DiHi	0.144	0.131	0.001	0.799	BA	Ca	En	2, 4, 6, 7, 9, 10
*Filicrisia geniculata* (Milne Edwards, 1838)	FiGe	0.002	0.000	0.002	0.002	B	Ca	Er	3
*Oncousoecia diastoporides* (Norman, 1869)	OnDi	0.024	0.021	0.003	0.045	BA	Ca	En	4, 10
*Oncousoecia polygonalis* (Kluge, 1915)	OnPo	0.005	0.000	0.005	0.005	A	Ca	En	10
*Patinella verrucaria* (Linnaeus, 1758)	PaVe	0.007	0.002	0.001	0.028	BA	Ca	En	4, 6, 7, 8, 9, 10, 12, 13, 15, 16, 17, 18
*Tubulipora flabellaris* (O. Fabricius, 1780)	TuFl	0.012	0.006	0.001	0.033	BA	Ca	En	4, 6, 10, 13, 15, 17
*Tubulipora ventricosa* (Busk, 1855)	TuVe	0.003	0.000	0.003	0.003	BA	Ca	Er	10
Ctenostomatida									
*Alcyonidioides mytili* (Dalyell, 1848)	AlMy	0.010	0.004	0.001	0.035	BA	Fl	En	3, 8, 9, 10, 13, 14, 17
*Alcyonidium gelatinosum diaphanum* (Farre, 1837)	AlGD	0.339	0.244	0.095	0.582	BA	Fl	Er	5, 18
*Alcyonidium hirsutum* (Fleming, 1828)	AlHi	0.481	0.428	0.003	3.050	BA	Fl	En	7, 8, 9, 10, 11, 12, 14
*Amathia arctica* (Busk, 1880)	AmAr	0.007	0.004	0.001	0.018	A	Fl	Er	3, 9, 10, 12
*Amathia imbricata* (Adams, 1800)	AmIm	0.044	0.035	0.001	0.250	B	Fl	Er	3, 8, 10, 12, 13, 14, 15
Cheilostomatida									
*Aquiloniella scabra* (van Beneden, 1848)	AqSc	0.488	0.000	0.488	0.488	BA	Ca	Er	10
*Bidenkapia spitzbergensis* (Bidenkap, 1897)	BiSp	0.002	0.001	0.001	0.004	BA	Ca	En	8, 13, 15
*Callopora craticula* (Alder, 1856)	CaCr	0.008	0.003	0.002	0.017	BA	Ca	En	3, 4, 5, 6, 7, 8, 9, 10, 11, 12, 13, 14, 15, 16
*Callopora lata* (Kluge, 1907)	CaLa	0.005	0.000	0.005	0.005	A	Ca	En	16
*Callopora lineata* (Linnaeus, 1767)	CaLi	0.022	0.007	0.001	0.100	BA	Ca	En	2, 3, 4, 6, 8, 9, 10, 12, 13, 14, 15, 16, 17
*Callopora weslawski* (Kuklinski and Taylor, 2006)	CaWe	0.005	0.001	0.001	0.009	A	Ca	En	3, 9, 10, 12, 13, 14, 17
*Celleporella hyalina* (Linnaeus, 1767)	CeHy	0.132	0.071	0.001	1.077	BA	Ca	En	1, 2, 3, 4, 6, 7, 8, 9, 10, 12, 13, 14, 15, 16, 17
*Cheilopora sincera* (Smitt, 1868)	ChSi	0.018	0.000	0.018	0.018	BA	Ca	En	10
*Copidozoum smitti* (Kluge, 1946)	CoSm	0.029	0.021	0.003	0.090	A	Ca	En	3, 8, 9, 10
*Cribrilina spitzbergensis* (Norman, 1903)	CrSp	0.079	0.032	0.001	0.332	A	Ca	En	3, 4, 8, 9, 12, 13, 14, 15, 17, 18
*Cylindroporella tubulosa* (Norman, 1868)	CyTu	0.008	0.004	0.001	0.034	BA	Ca	En	3, 4, 8, 9, 10, 13, 16, 17
*Dendrobeania fruticosa* (Packard, 1863)	DeFr	0.312	0.220	0.060	0.970	BA	Ca	Er	4, 6, 10, 15
*Dendrobeania levinseni* (Kluge, 1929)	DeLe	0.288	0.000	0.288	0.288	BA	Ca	Er	13
*Dendrobeania murrayana* (Bean in Johnston, 1847)	DeMu	0.449	0.444	0.005	0.893	BA	Ca	Er	10, 14
*Dendrobeania quadridentata* (Lovén, 1834)	DeQu	0.113	0.000	0.113	0.113	BA	Ca	Er	10
*Doryporella spathulifera* (Smitt, 1868)	DoSp	0.002	0.000	0.002	0.002	BA	Ca	En	2
*Einhornia arctica* (Borg, 1931)	EiAr	0.027	0.008	0.001	0.078	BA	Ca	En	3, 4, 6, 8, 9, 10, 12, 13, 14, 15, 16, 17
*Eucratea loricata* (Linnaeus, 1758)	EuLo	1.203	0.677	0.006	7.570	BA	Ca	Er	3, 4, 7, 8, 9, 10, 12, 13, 14, 15, 18
*Harmeria scutulata* (Busk, 1855)	HaSc	0.086	0.056	0.001	0.862	A	Ca	En	1, 3, 4, 6, 7, 8, 9, 10, 12, 13, 14, 15, 16, 17, 18
*Hippoporella hippopus* (Smitt, 1868)	HiHi	0.010	0.002	0.006	0.014	BA	Ca	En	2, 4, 10
*Juxtacribrilina annulata* (Fabricius, 1780)	JuAn	0.003	0.001	0.001	0.006	BA	Ca	En	1, 6, 7, 15, 16, 17
*Lepraliella contigua* (Smitt, 1868)	LeCo	0.004	0.003	0.001	0.006	BA	Ca	En	2, 11
*Microporella arctica* (Norman, 1903)	MiAr	0.076	0.051	0.007	0.479	B	Ca	En	2, 3, 4, 5, 6, 7, 8, 9, 10, 11, 12, 13, 14, 15
*Porella acutirostris* (Smitt, 1868)	PoAc	0.020	0.013	0.004	0.047	BA	Ca	En	9, 10, 13
*Porella aperta* (Boeck, 1862)	PoAp	0.006	0.000	0.006	0.006	A	Ca	En	10
*Porella belli* (Dawson, 1859)	PoBe	0.003	0.000	0.003	0.003	BA	Ca	En	4
*Porella concinna* (Busk, 1854)	PoCo	0.020	0.016	0.003	0.052	BA	Ca	En	4, 10, 12
*Ragionula rosacea* (Busk, 1856)	RaRo	0.044	0.000	0.044	0.044	BA	Ca	Er	10
*Rhamphostomella plicata* (Smitt, 1868)	RhPl	0.008	0.000	0.008	0.008	BA	Ca	En	10
*Rhamphostomella scabra* (O. Fabricius, 1824)	RhSc	0.018	0.000	0.018	0.018	BA	Ca	En	10
*Rhamphostomella spinigera* (Lorenz, 1886)	RhSp	0.006	0.000	0.006	0.006	A	Ca	En	10
*Schizomavella (Schizomavella) auriculata* (Hassall, 1842)	ScAu	0.107	0.100	0.007	0.207	B	Ca	En	10, 14
*Schizomavella (Schizomavella) lineata* (Nordgaard, 1896)	ScLi	0.061	0.042	0.019	0.102	BA	Ca	En	10, 12
*Schizomavella porifera* (Smitt, 1868)	ScPo	0.001	0.000	0.001	0.001	BA	Ca	En	4
*Schizoporella costata* (Kluge, 1962)	ScCo	0.006	0.004	0.002	0.010	BA	Ca	En	10, 15
*Smittina minuscula* (Smitt, 1868)	SmMi	0.001	0.000	0.001	0.001	BA	Ca	En	10
*Smittina smitti* (Kirchenpauer, 1874)	SmSm	0.025	0.000	0.025	0.025	A	Ca	En	10
*Stomacrustula cruenta* (Busk, 1854)	StCr	0.005	0.002	0.003	0.006	BA	Ca	En	4, 12
*Stomacrustula pachystega* (Kluge, 1929)	StPa	0.012	0.000	0.012	0.012	BA	Ca	En	10
*Tegella arctica* (d’Orbigny, 1853)	TeArc	0.073	0.024	0.003	0.359	BA	Ca	En	2, 3, 4, 6, 7, 8, 9, 10, 12, 13, 14, 15, 16, 17, 18
*Tegella armifera* (Hincks, 1880)	TeArm	0.010	0.003	0.004	0.019	BA	Ca	En	9, 10, 16, 17
*Tegella retroversa* (Kluge, 1952)	TeRe	0.043	0.023	0.002	0.129	BA	Ca	En	4, 8, 9, 10, 12
*Tricellaria elongata* (Smitt, 1868)	TrEl	0.039	0.035	0.004	0.074	A	Ca	Er	10, 15
*Tricellaria gracilis* (Van Beneden, 1848)	TrGr	0.667	0.375	0.001	3.123	BA	Ca	Er	4, 9, 10, 12, 13, 14, 15, 16, 17
*Tricellaria ternata* (Ellis and Solander, 1786)	TrTe	0.544	0.191	0.002	1.443	BA	Ca	Er	3, 9, 10, 11, 12, 13, 14, 15
*Turbicellepora incrassata* (Lamarck, 1816)	TuIn	4.360	0.000	4.360	4.360	A	Ca	Er	10

Note: X—mean, SE—standard error, Min—minimum, Max—maximum, Or—origin (B—boreal species, BA—Boreo-Arctic species, A—Arctic species), CF—construction forms (Ca—calcified, Fl—flexible), OF—orientation forms (Er—erect, En—encrusting).

**Table 2:** Results of SIMPER analysis: contribution of bryozoan species to the total dissimilarity between the groups delineated with cluster analysis in Grønfjorden.

Species	Average Dissimilarity, %	Contribution, %	Cumulative Contribution, %
*Eucratea loricata*	6.05	9.49	9.49
*Tricellaria ternata*	4.99	7.82	17.30
*Tricellaria gracilis*	3.58	5.61	22.92
*Alcyonidium hirsutum*	3.58	5.60	28.52
*Cribrilina spitzbergensis*	3.41	5.35	33.87
*Microporella arctica*	2.87	4.50	38.38
*Crisiella diversa*	2.52	3.95	42.33
*Einhornia arctica*	2.25	3.53	45.86
*Callopora lineata*	2.24	3.50	49.37
*Amathia imbricata*	2.15	3.36	52.73
*Celleporella hyalina*	2.10	3.29	56.02
*Alcyonidioides mytili*	1.64	2.58	58.59
*Dendrobeania fruticosa*	1.61	2.52	61.12
*Disporella hispida*	1.55	2.43	63.54
*Harmeria scutulata*	1.52	2.39	65.93
*Juxtacribrilina annulata*	1.49	2.34	68.27
*Crisiella producta*	1.46	2.30	70.57

**Table 3:** List of environmental variables contributed to the RDA models based on the bryozoan diversity and biomass data in Grønfjorden.

Variable	Diversity				Biomass		
	LambdaA	F	*p*	Variable	LambdaA	F	*p*
T	21	12.32	0.001	T	13	2.39	0.042
Macrophytes	17	3.19	0.081	Macrophytes	8	2.08	0.030
Gravel	13	2.80	0.099	Depth	6	1.43	0.187
Pebble	3	1.50	0.227	Gravel	5	1.24	0.274
Sand	1	0.59	0.624	S	4	1.09	0.355
Boulders	1	0.68	0.581	Sand	4	0.83	0.525
S	1	0.47	0.712	Pebble	3	1.02	0.416

Note: T—temperature (°C), S—salinity, Sand, Gravel, Pebbles, Boulders, Macrophytes—proportion of corresponding substrate. LambdaA—explained variation, %, F—pseudo F-ratio, *p*—probability level.

## Data Availability

The data are available upon request from the corresponding author.
